# Takotsubo cardiomyopathy in a Caucasian Italian woman: Case report

**DOI:** 10.1186/1476-7120-5-18

**Published:** 2007-04-06

**Authors:** Matteo Lisi, Valerio Zacà, Silvia Maffei, Francesca Casucci, Marianna Maggi, Stefano Lunghetti, Paolo Aitiani, Arcangelo Carrera, Debora Castellani, Roberto Favilli, Carlo Pierli, Sergio Mondillo

**Affiliations:** 1 Department of Cardiology, University of Siena, Viale Bracci 1, 53100, Siena, Italy

## Abstract

**Background:**

Takotsubo cardiomyopathy is an acute cardiac syndrome characterized by transient LV regional wall motion abnormalities (with peculiar apical ballooning appearance), chest pain or dyspnea, ST-segment elevation and minor elevations of cardiac enzyme levels

**Case presentation:**

A 68-year-old woman was admitted to the Emergency Department because of sudden onset chest pain occurred while transferring her daughter, who had earlier suffered a major seizure, to the hospital. The EKG showed sinus tachycardia with ST-segment elevation in leads V2–V3 and ST-segment depression in leads V5–V6, she was, thus, referred for emergency coronary angiography. A pre-procedural transthoracic echocardiogram revealed regional systolic dysfunction of the LV walls with hypokinesis of the mid-apical segments and hyperkinesis of the basal segments. Coronary angiography showed patent epicardial coronary arteries; LV angiography demonstrated the characteristic morphology of apical ballooning with hyperkinesis of the basal segments and hypokinesis of the mid-apical segments. The post-procedural course was uneventful; on day 5 after admission the echocardiogram revealed full recovery of apical and mid-ventricular regional wall-motion abnormalities.

**Conclusion:**

Takotsubo cardiomyopathy is a relatively rare, unique entity that has only recently been widely appreciated. Acute stress has been indicated as a common trigger for the transient LV apical ballooning syndrome, especially in postmenopausal women. The present report is a typical example of stress-induced takotsubo cardiomyopathy in a Caucasian Italian postmenopausal woman.

## Background

Takotsubo cardiomyopathy, also known as transient left ventricular (LV) apical ballooning syndrome, is an acute cardiac syndrome characterized by transient LV regional wall motion abnormalities, chest pain or dyspnea, ST-segment elevation and minor elevations of cardiac enzyme levels [1]. The typical feature of the syndrome is a transient regional systolic dysfunction involving the LV apex and mid-ventricle with concomitant hyperkinesis of the basal LV segments [1]. The syndrome has been first described in the Japanese population [2] and subsequently in the Caucasian population in both Europe [3] and U.S. [4], and was named takotsubo after a round bottomed narrow-necked Japanese fishing pot used for trapping octopus.

## Case presentation

**Figure 1:**
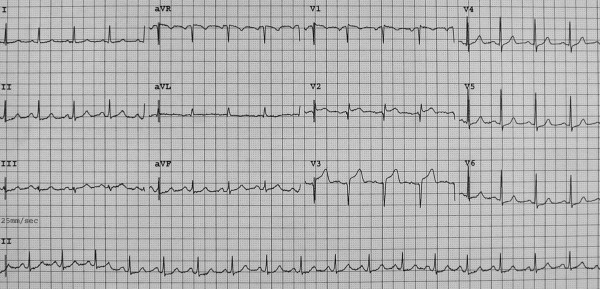
**Twelve-lead electrocardiogram on admission**. Sinus tachycardia with 2–3 mm ST-segment elevation in leads V2–V3 and 1 mm ST-segment depression in leads V5–V6.

**Figure 2:**
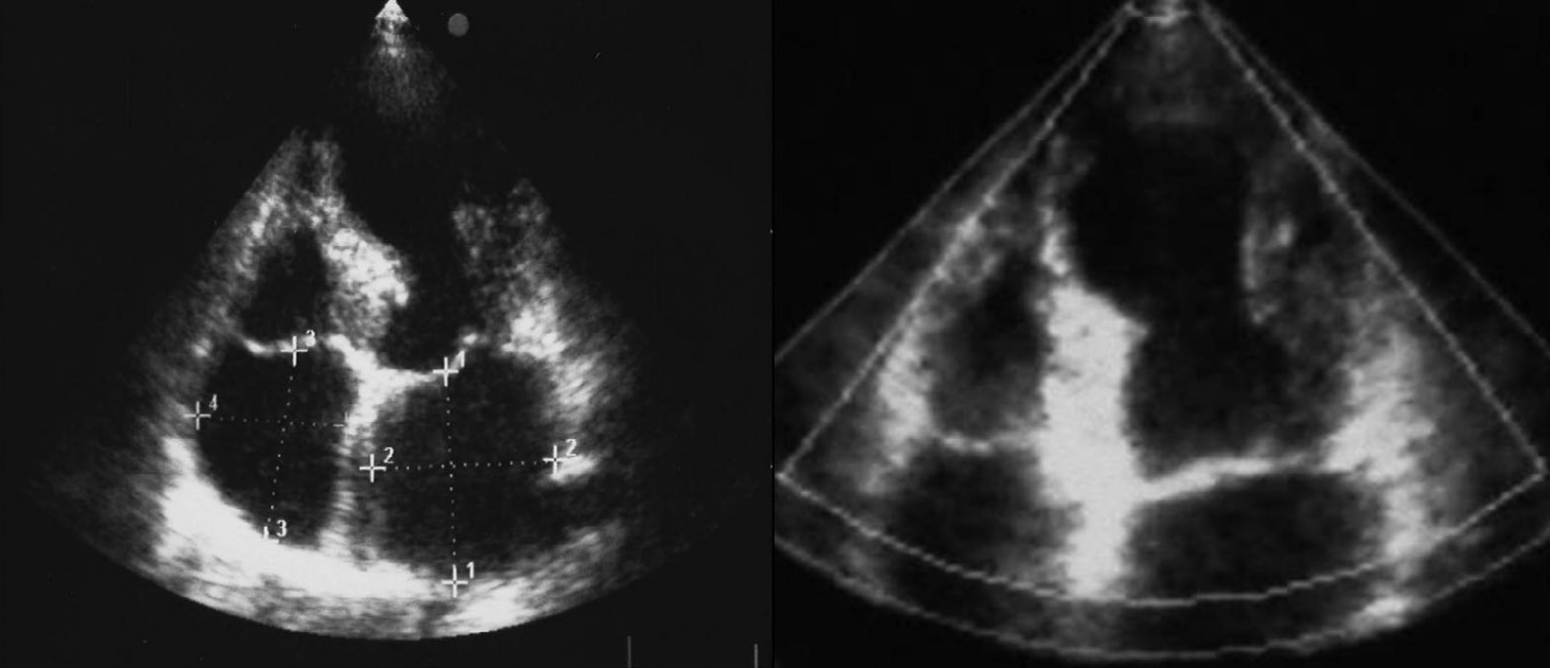
**Transthoracic echocardiogram on admission**. A. Apical 4-chamber view showing apical ballooning of the left ventricle. B. Zoom detail of the same view as in A.

**Figure 3:**
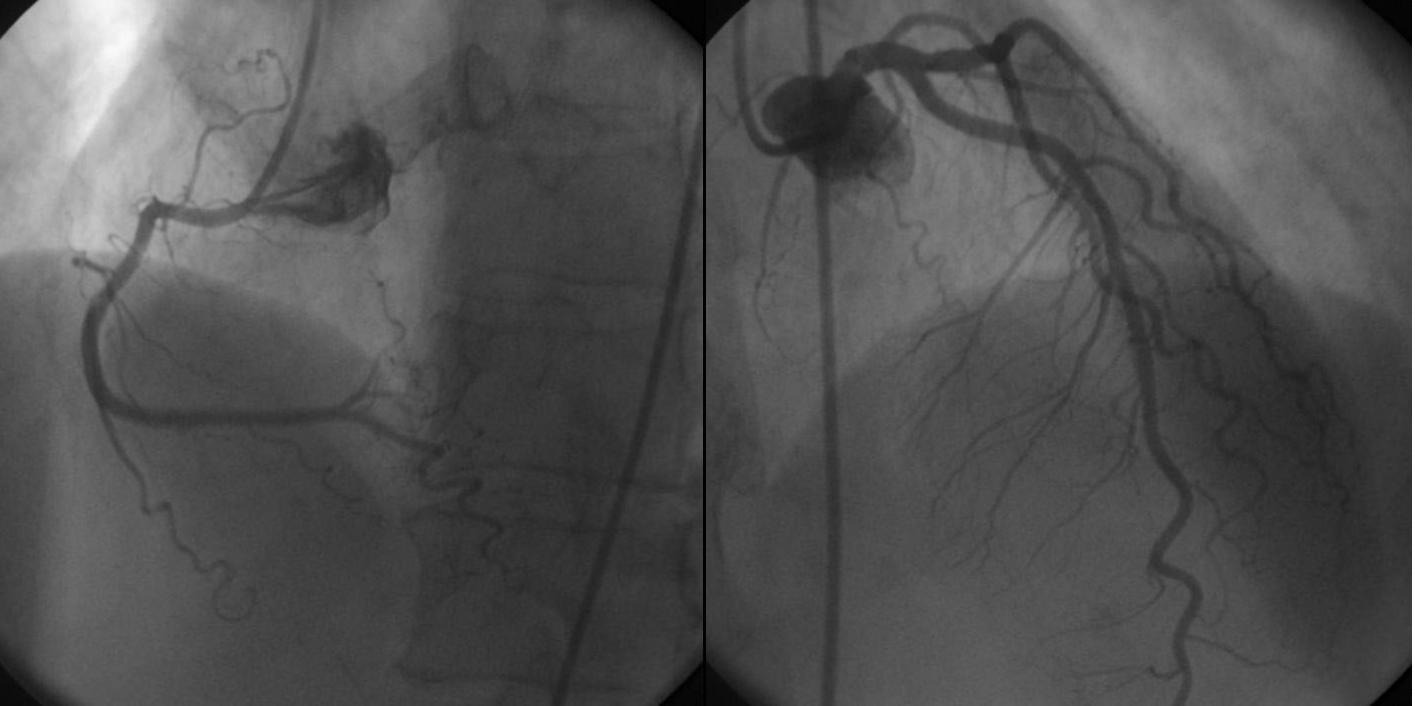
**Coronary angiography**. Selective right coronary artery angiography (A) and left coronary artery angiography (B) demonstrating no angiographically detectable coronary artery disease.

**Figure 4:**
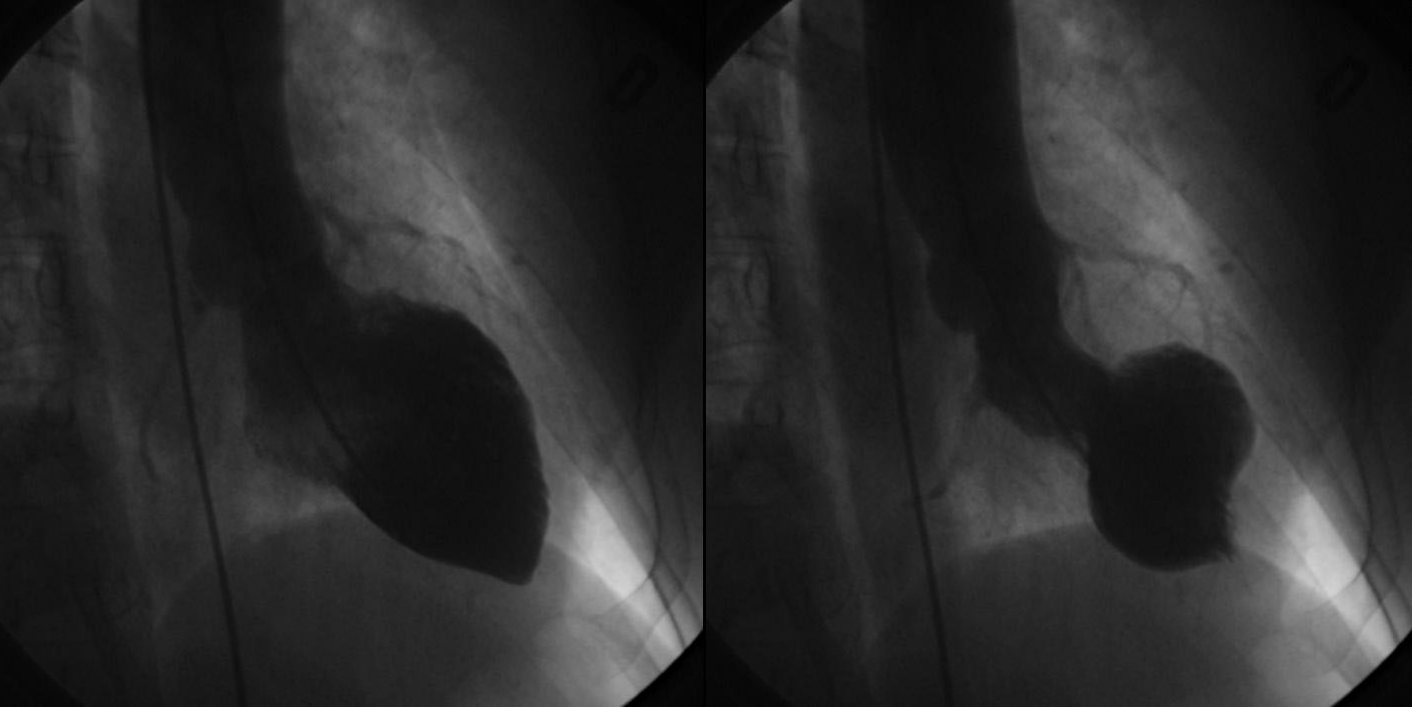
**Left ventriculograms**. Diastolic (A) and systolic (B) morphology of the left ventricle with the typical appearance of apical ballooning in systole.

**Figure 5:**
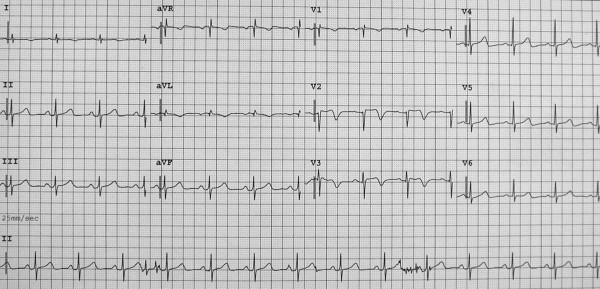
**Twelve-lead electrocardiogram on day 1 after admission**. Evolutionary T wave inversion in leads V2–V3 with ST-segment normalization in leads V5–V6.

**Figure 6:**
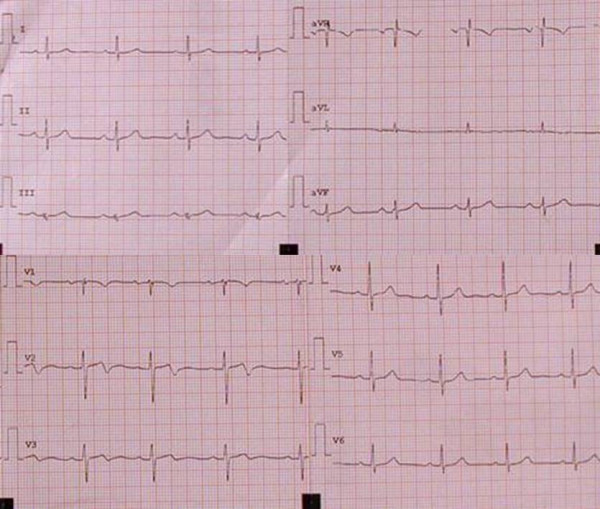
**Twelve-lead electrocardiogram on day 5 after admission**. Persistent T wave inversion in leads V2–V3.

**Figure 7:**
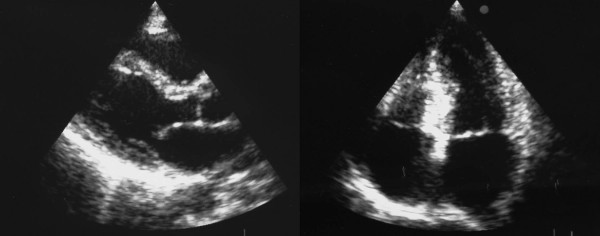
**Transthoracic echocardiogram on day 5 after admission**. Parasternal long-axis view (A) and apical 4-chamber view (B) showing recovery of wall-motion abnormalities with disappearance of apical ballooning.

A 68-year-old woman was admitted to the Emergency Department because of sudden onset chest pain occurred while transferring her daughter, who had earlier suffered a major seizure, to the hospital. Her cardiovascular risk factors were female sex, tobacco smoking, dyslipidemia and family history of coronary artery disease. Upon admission her blood pressure was 150/80 mmHg and the electrocardiogram (EKG) showed sinus tachycardia with 2–3 mm ST-segment elevation in leads V2–V3 and 1 mm ST-segment depression in leads V5–V6 (Figure 1). She was, thus, diagnosed with ST-elevation acute myocardial infarction and referred for emergency coronary angiography. A pre-procedural transthoracic echocardiogram revealed regional systolic dysfunction of the LV walls with hypokinesis of the mid-apical segments and hyperkinesis of the basal segments with ejection fraction (EF) of 40% (Figure 2A and 2B). Coronary angiography showed patent epicardial coronary arteries with no evidence of spasm or thrombosis and only minor atherosclerotic manifestations (Figure 3A and 3B); LV angiography demonstrated the characteristic morphology of apical ballooning with hyperkinesis of the basal segments and hypokinesis of the mid-apical segments (Figure 4A and 4B). Provocative tests for induction of coronary vasospasm were not performed. The patient was then transferred to the coronary intensive care unit for post-procedural continuous monitoring and started on oral aspirin, diltiazem, ramipril, atorvastatin and sub-cutaneous low molecular weight heparin. Results of laboratory analysis showed a peak serum Troponin T level of 0.44 ng/ml (normality range 0.06–0.1). EKG on day 1 after admission showed evolutionary T wave inversion in leads V2–V3 with ST-segment normalization in leads V5–V6 (Figure 5). On day 5 after admission the EKG showed persistent T wave inversion in leads V2–V3 (Figure 6), while the echocardiogram revealed full recovery of apical and mid-ventricular regional wall-motion abnormalities with normal EF (Figure 7A and 7B). The post-procedural course was uneventful; takotsubo cardiomyopathy was the final diagnosis and the patient was, thus, discharged with a therapy consisting of aspirin, diltiazem, ramipril and atorvastatin.

## Conclusion

Takotsubo cardiomyopathy is a relatively rare, unique entity that has only recently been widely appreciated [1]. Although the exact cause of the syndrome remains unknown, many underlying mechanisms have been, so far, proposed including diffuse epicardial arteries spasm, coronary microcirculation dysfunction, cathecolamines-induced myocardial dysfunction, and neurologically-mediated myocardial stunning [1]. Acute stress has been indicated as a common trigger for the transient LV apical ballooning syndrome [1,5]. Interestingly, it has been reported that the majority of patients experiencing the syndrome were post-menopausal Japanese women who present ischemic-like chest pain early after an episode of acute emotional or physiologic stress [1,5]. In general most patients were women (ranging from 82 to 100% in different series) with a mean age at presentation of approximately 70 years [1]. Explanation for this dramatic sex and age discrepancy can only be speculated, however it may be possibly related to post-menopausal alterations of endothelial function secondary to reduced estrogen levels and microcirculatory vasomotor reactivity to cathecolamine-mediated stimuli [1]. Initially, takotsubo cardiomyopathy was believed to have a peculiar geographic and racial distribution given the predilection for Japanese women and the lack of reports of case-series from other countries [1,2]. Desmet et al. in 2003 first described the syndrome in a series of 13 Caucasian patients from Belgium [3]; more recently other groups from both North America and Europe reported series of LV apical ballooning in white women, the largest, by Sharkey et al., involving 22 subjects [1,4,6,7]. The present case is a typical example of stress-induced takotsubo cardiomyopathy in a Caucasian Italian postmenopausal woman.

## Competing interests

The author(s) declare that they have no competing interests.

## Authors' contributions

**ML **collected the data relative to the Case Report; **VZ **assisted the patient in the CICU and composed the draft of the manuscript; **SM **participated in the preparation of the revised manuscript; **FC **collected the data relative to the Case Report; **MM **collected the data relative to the Case Report; **SL **performed the first echocardiogram; **PA **performed the second echocardiogram; **AC **performed the coronary angiography; **DC **helped in revising the manuscript draft; **RF **is head of the CICU and participated in the coordination and revision of the manuscript; **CP **is head of the Cath Lab and participated in the coordination and revision of the manuscript; **SM **is head of the Echo Lab and conceived the Case report and participated in the coordination, data analysis and elaboration and drafting of the manuscript. All authors read and approved the final manuscript.
